# Vertically Conductive Single-Crystal SiC-Based Bragg Reflector Grown on Si Wafer

**DOI:** 10.1038/srep17026

**Published:** 2015-11-25

**Authors:** David Massoubre, Li Wang, Leonie Hold, Alanna Fernandes, Jessica Chai, Sima Dimitrijev, Alan Iacopi

**Affiliations:** 1 Griffith University, Queensland Micro and Nanotechnology Centre, Brisbane, 4111, Australia; 2 Bluglass Ltd., Sydney, 2128, Australia

## Abstract

Single-crystal silicon carbide (SiC) thin-films on silicon (Si) were used for the fabrication and characterization of electrically conductive distributed Bragg reflectors (DBRs) on 100 mm Si wafers. The DBRs, each composed of 3 alternating layers of SiC and Al(Ga)N grown on Si substrates, show high wafer uniformity with a typical maximum reflectance of 54% in the blue spectrum and a stopband (at 80% maximum reflectance) as large as 100 nm. Furthermore, high vertical electrical conduction is also demonstrated resulting to a density of current exceeding 70 A/cm^2^ above 1.5 V. Such SiC/III-N DBRs with high thermal and electrical conductivities could be used as pseudo-substrate to enhance the efficiency of SiC-based and GaN-based optoelectronic devices on large Si wafers.

Growth of single-crystal silicon carbide on silicon substrate (SiC-on-Si) is seen as a very attractive approach to combine the excellent properties of SiC with the low cost, large wafer size and well-developed micro-machining of Si wafers. Despite their large lattice and thermal expansion mismatches, both around 20%, uniform and crack-free single-crystal SiC-on-Si templates can be obtained with a relatively good crystal quality^1^. Consequently, SiC-on-Si pseudo-substrate are now investigated for a broad range of applications including photonic^2^,^3^, gallium nitride based (GaN) devices on Si^4^,^5^, micro-electro-mechanical systems (MEMS)^6^ and graphene epitaxial growth^7^.

We propose and demonstrate for the first time the use of the SiC-on-Si technology to fabricate a vertically conductive single-crystal distributed Bragg reflector (DBR) on Si substrate. Such SiC-based DBRs enable the monolithic integration of efficient GaN-based optoelectronic devices on large Si wafers. SiC is indeed commonly used as growth substrate for commercial high power GaN devices as it has the smallest lattice mismatch amongst all foreign substrates for the hetero-epitaxy of III-nitride compound semiconductors, typically less than 4%. However, as SiC substrates are smaller and much more expensive than Si substrates, the SiC-on-Si technology is economically very attractive for the monolithic integration of GaN devices on large Si wafers. Several demonstrations of GaN light emitting diodes (GaN-LEDs) and GaN power devices grown on SiC-on-Si substrates have already been reported^4^,^5^. By using Si as a platform technology, GaN devices can also be directly integrated with CMOS devices and depreciated CMOS factory plants can be utilized, which lead to substantial cost saving in capital equipment investment and device fabrication costs. Thanks to its high electrical and thermal conductivities, combined with a large refractive index (RI) and a low absorption in the visible spectrum^8^, SiC is also a material of choice for the fabrication of single-crystal DBRs on Si operating in the visible or the infrared (IR) spectra. Light extraction (or absorption) efficiency of an optoelectronic device can be greatly improved with a DBR when used as a rear mirror sandwiched between the Si substrate and the device structure^9^. It is therefore very attractive to develop a SiC-based DBR for GaN-LEDs on Si. Another advantage of monolithic DBR-LEDs on Si would be to greatly simplify the device processing, and thus to reduce the manufacturing cost, compared to the standard GaN-LED on Si technology. Indeed, because of the strong optical absorption occurring in the Si substrate, manufacturing of high brightness (HB) LEDs on Si currently requires the removal of the Si growth substrate followed by the transfer of the III-N epilayers to a new high reflective carrier^10^. This process is particularly difficult and expensive to apply on large substrates as it requires low wafer bowing and often expensive gold-based bonding layer, hence lowers the process yield and induces a high manufacturing cost.

A DBR consists of multiple transparent layers with alternative high and low RI, and with each layer thickness carefully chosen to create an optical resonance effect at the desired wavelength^11^. DBRs are fundamental for the fabrication of many photonic and optoelectronic devices using optical resonance effects in a microcavity^12^. Such devices include Fabry-Perot filters and modulators, resonant cavity (RC)-LEDs and vertical cavity surface emitting lasers (VCSELs). Monolithic growth of a DBR for GaN devices on Si requires the use of transparent materials which are compatible with both Si and III-N semiconductors, strongly limiting the number of suitable candidates. Most of the demonstrations of DBR for GaN-devices (mainly targeting LED applications) on Si have been made using only III-N semiconductors as the constitutive layers^13^. Reflectance as high as 95% was achieved, but as many as 20 pairs AlInN/GaN were needed because of the small RI contrast achievable between those III-N semiconductor layers^14^. Growth of such thick stack of layers implies also the use of complex stress management during the heteroepitaxy on Si because of the large lattice mismatch, making it challenging to grow crack-free DBR-LEDs. So far, the only successful report of such DBR-LED on Si was achieved only by using DBRs with a small number of pairs and thus with a relatively low reflectance^15^. Another drawback with III-N DBRs comes from their weak thermal and electrical conductivities which strongly limit their attractiveness for HB-LEDs. Rare-earth-oxides based DBRs paired with Si thin-films have also been investigated as the high RI contrast allows high reflectivity and large stopband in the visible with just few pairs^16^,^17^. However, their detrimental thermal and electrical properties strongly limit their potential for HB-LEDs as well^18^.

In this paper, vertically conductive DBRs, using single-crystal SiC thin-films paired with doped or undoped Al(Ga)N layers, were heteroepitaxially grown on large Si substrates. High uniformity over 100 mm Si wafers, with a typical average peak reflectance of ~55% centered in the blue wavelengths and a stopband of 100 nm, is demonstrated using 1.5 DBR pairs. Furthermore, the DBR structure using Si-doped AlGaN shows very good vertical electrical conductivity, with current density as high as 70 A/cm^2^ at 1.5 V, without visible degradation of the optical performance compared to its non-conductive counterpart. Such DBR structures with high electrical conductivity of materials with high thermal conductivity are ideal candidates for the monolithic integration of SiC-based and GaN-based high power optoelectronic devices on Si.

## Design of the SiC-based DBRs on Si

### Advantages of the SiC-on-Si pseudo-substrate

**Figure 1:**
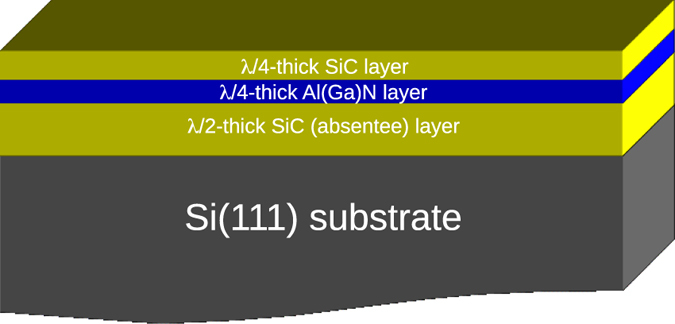
Schematic of a SiC/Al(Ga)N DBR structure on Si substrate.

The DBR structures are composed of a half-wavelength (λ/2)-thick SiC layer, followed by 1 pair of quarter-wavelength (λ/4)-thick AlN/SiC layers (DBR A) or AlGaN/SiC layers (DBR B) as illustrated in Fig. 1. Starting the DBR growth with a SiC layer, instead of directly with the III-N layer, enables the circumvention of several drawbacks of the III-N growth on Si. Firstly, a III-N deposition on SiC should yield an higher crystalline quality than directly on Si thanks to a lower lattice mismatch of 3% compared to 17%^10^. In addition, stress management for the whole heteroepitaxy should also be easier to engineer due to the lower lattice and thermal mismatch. Secondly, an important advantage of the SiC template is to protect the Si substrate from the “melt-back etching” that would otherwise occur due to the formation of a low temperature eutectic between Ga and Si and thus eliminating the need of the usual protective AlN buffer layer with its detrimental current blocking effect. Indeed, AlN is not only intrinsically highly resistive but also induces a large band discontinuity at the Si interface strongly impairing the vertical current flow^19^. In other words, by starting the DBR growth with a highly conductive SiC layer, it can both protect the Si substrate from reacting with Ga and provide a high electrical conductivity for vertical current injection. Vertical current injection is important because it is the most efficient current scheme for optoelectronic devices such as GaN-LEDs^20^. As SiC has a higher refractive index than Al(Ga)N, the SiC template thickness must be equal to λ/2 to avoid an anti-reflection effect and thus acts as an absentee layer^11^. We emphasize that because of the temperature limitation imposed by the melting point of Si at 1420 °C, only the cubic crystalline structure of SiC, called cubic polytype (or 3C-SiC), can be grown on Si with the LPCVD technique as used in this work^1^.

### Optical constants

**Figure 2:**
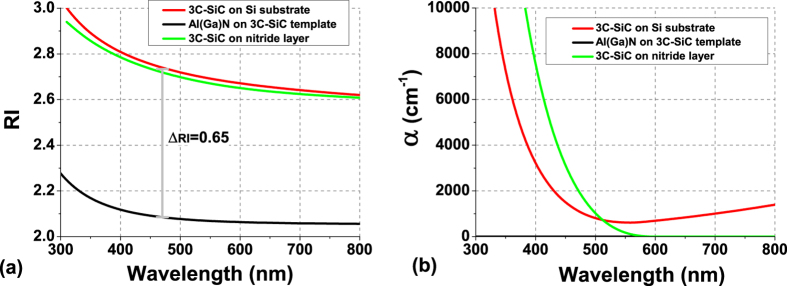
Dispersion of the refractive indices (RI) (**a**) and coefficients of absorption (α) (**b**) for the single-crystal layers constituting the DBRs on Silicon substrate.

Design of a DBR structure requires the knowledge of the optical constants of each constitutive layer. Figure 2(a,b) show the dispersion, deduced from spectroscopy ellipsometry, of RI and of the coefficient of absorption (α) respectively, for the 3C-SiC and nitride thin-films. Data for each material were averaged from fitting made on several test samples and then used to optimise the design of the SiC-based DBRs. AlN and AlGaN layers gave very similar ellipsometric results with no absorption over the investigated spectral range and the dispersion of RI is similar to reported values for AlN thick-films grown on bulk SiC^21^, indicating that a good crystal quality nitride layer has been grown on the SiC-on-Si pseudo-substrate. For convenience, the same RI dispersion for both nitride materials deposited on the SiC template was chosen. 3C-SiC layers grown on Si or on a nitride layer gave similar dispersion of RI, with values in good agreement with reported data for 3C-SiC material^22^. As a result, between 3C-SiC and Al(Ga)N, one obtains a large RI contrast of 0.65 in the blue spectrum which is already 2 to 3 times larger compared to values obtained with the standard pure nitride-based DBRs^9^. Analysis of the optical absorption shows a strong difference between 3C-SiC layers grown on Si and Al(Ga)N. For SiC deposited on Si, there is a significant and increasing residual absorption above 500 nm, whereas SiC deposited on Al(Ga)N show no detectable absorption above 550 nm. As 3C-SiC has an indirect bandgap of ~2.4 eV, any absorption below this energy (i.e. above 520 nm) is induced by sub-bandgap traps created by the crystalline defects. Because of the large lattice mismatch, SiC layers grown on Si have large density of defects, up to 10^10^ cm^−3^, leading to the residual absorption detected on the SiC on Si layers^1^. On the other hand, the lower lattice mismatch between SiC and III-N semiconductors is expected to provide much better crystalline quality resulting in a lower residual absorption as seen in our SiC grown on nitride layer.

## DBRs Characterization and Discussion

### Structural Characterization

**Figure 3:**
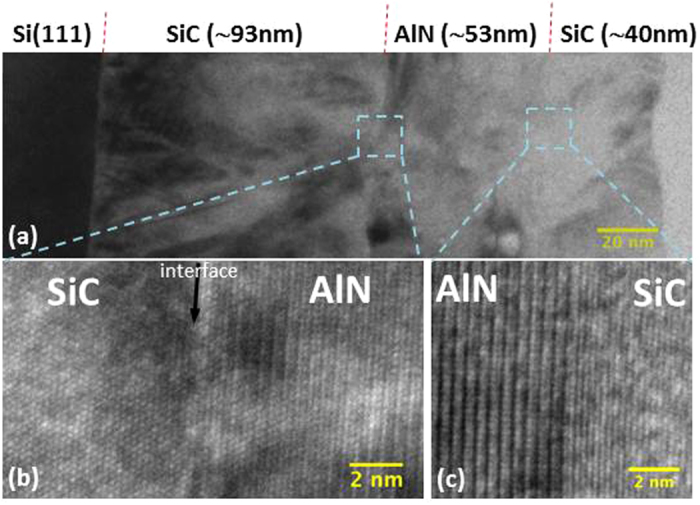
Bright field (**a**) and high resolution (**b,c**) TEM images of a cross-section 1.5 pairs SiC/AlN DBR structure on Si. Images (**b**,**c**) correspond respectively to the SiC/AlN and AlN/SiC interfaces.

**Figure 4:**
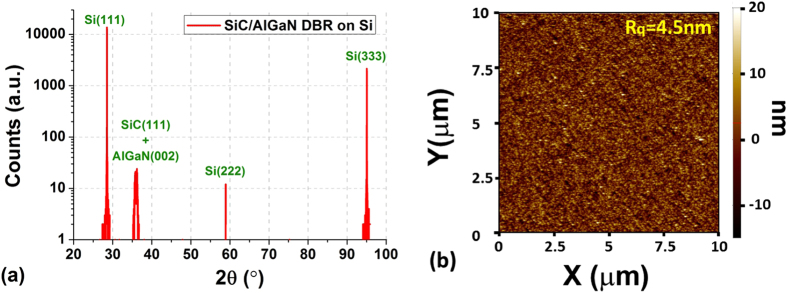
(**a**) XRD 2θ-ω locked scan of SiC/AlGaN DBR structure on Si(111) and (**b**) AFM image of a 10 μm × 10 μm area from the SiC/AlGaN DBR surface.

Figure 3 shows typical bright field and high-resolution TEM images taken at the cross-section of the DBR A. As expected, a high density of defects, composed mostly of stacking faults and misfit dislocations, can be seen propagating from the Si/SiC interface (*cf.* Fig. 3(a)), while a much smoother transition is seen at both SiC/nitride interface. Interestingly, dislocations generated at the Si/SiC interface do not seem to propagate into the upper layers, which explains the good optical quality (i.e. low residual absorption) of those layers grown on top of the SiC-on-Si template. In addition, Fig. 3(b,c) show that the interface is coherent and single crystal, which is due to the low lattice mismatch between the two layers. It is worth noting that no Si voids were detected by TEM near the Si/SiC interface, thanks to the optimization of our SiC deposition process^23^. Such voids would be detrimental for optoelectronic devices by impairing thermal dissipation and electrical conduction^1^. A typical XRD 2θ-ω scan for the DBR B, displayed in Fig. 4(a), confirms that the structure is composed only of single-crystal layers. Indeed, beside the 3 diffraction peaks caused by the Si(111) substrate, only one additional peak can be detected, which is in fact a combination of the SiC(111) peak (~35.8°) and the AlGaN(002) peak (~36.1°) as confirmed by a high-resolution ω-2θ XRD scan (not shown here). We obtain similar results for the DBR A but with an AlN(002) diffraction peak at ~36.2°. From the ω-2θ XRD scan, it was estimated that the Ga concentration in the AlGaN layer composing the DBR B was about 6% ± 1%. This is in a good agreement with XPS measurement, which gave an atomic ratio of 4% to 5% for Ga. Figure 4(b) shows a typical AFM scan image from the top surface of the DBR B. The root mean square roughnesses (R_*q*_), averaged from multiple random scans of 10 μm × 10 μm over each wafer, were equivalent and equal to 4.5 nm for all DBR structures. While a smaller roughness is always preferable to serve as a better template for subsequent epitaxial growth, high quality III-N hetero-structures have successfully been grown using SiC-on-Si templates with similar roughness^24^,^25^. Finally, the variation of wafer curvature following the stack deposition was measured and shows a similar convex-shaped change for both types of DBR, demonstrating a residual tensile stress at room temperature as expected for SiC and AlGaN thin-film growths on Si^1^,^10^. However, the increase in bow height is kept relatively small with only +2.8 μm and +3.0 μm for DBR A and B respectively, thanks mainly to the epitaxial growth of SiC on both wafer sides compensating in part the induced stress. It is important to limit the change of wafer curvature as a high bow height would induce a non-uniform LED growth and also significant device processing issues^10^.

### Optical properties

**Figure 5:**
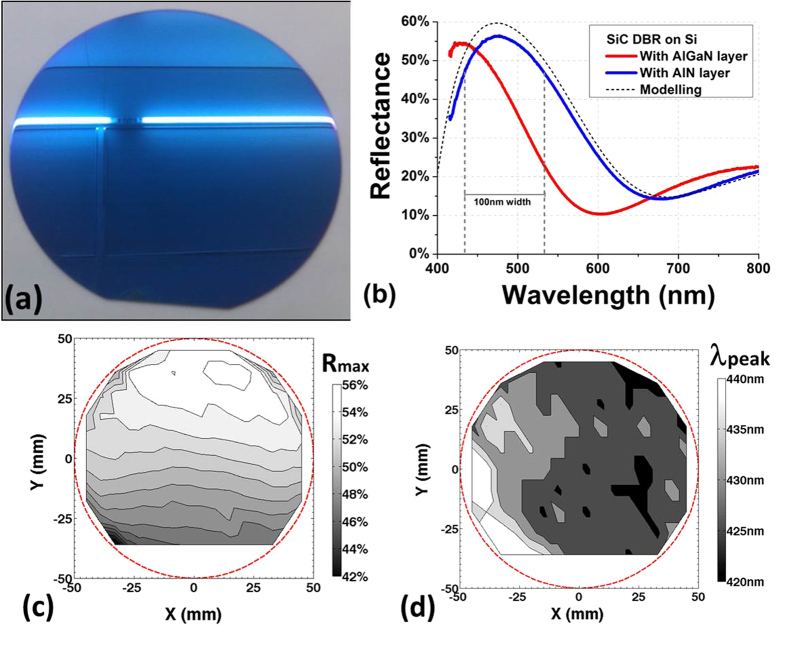
(**a**) Optical image of a 100 mm Si wafer coated with a 1.5 pairs SiC/AlGaN DBR structure. (**b**) Experimental and numerical reflectance spectra from SiC-based DBRs on Si. Mapping of the maximum reflectance R_*max*_ (**c**) and the corresponding wavelength λ(R_*max*_) (**d**) for the SiC/AlGaN DBR on Si wafer shown in (**a**). Mappings are made of 136 equally spaced measurements with an edge exclusion of few mm. The red dashed lines in (**c**,**d**) draw the circumference of a 100 mm-diameter wafer for eye guiding purpose.

Figure 5(a) shows a photograph of a crack-free 100 mm Si wafer coated with DBR B. One can see by naked eye a uniform blue color, as expected per design, over all the surface of the wafer which is a good indicator of the DBR uniformity. Typical reflectance spectra from DBRs A and B are shown in Fig. 5(b). Due to the detection limit from the reflectometer system, only the reflectance above 415 nm could be measured. DBR A shows a maximum reflectance R_*max*_ of 56.5% centered at λ(R_*max*_) = 475 nm, very close to the targeted peak wavelength of 470 nm, while DBR B shows a R_*max*_ value of 54.5% at a lower wavelength of 430 nm. This lower peak wavelength than expected is due to an error during the top SiC layer deposition resulting in a thinner SiC layer than planned. For comparison, pure-nitride DBRs require about 5 pairs to obtain comparable R_*max*_, mainly because of their much smaller RI contrast^15^,^26^. Furthermore, a large photonic stopband is also obtained with a width of ~100 nm at 80% of R_*max*_, which is 3 to 4 times larger than with pure nitride DBRs^9^. Such large stopband is important to improve the efficiency of optoelectronic devices with large spectrum of emission or detection such as white LEDs and photodetectors. In order to evaluate the quality of our SiC-based DBR structures, theoretical reflectance of DBR A was calculated using the transfer matrix method^11^ and is plotted with a black dashed line in Fig. 5(b) for comparison purpose. This numerical response, based on the experimental optical constants (*cf.* Fig. 2) and the layer thicknesses deduced from the TEM images (*cf.* Fig. 3(a)) shows a good agreement with the experimental reflectance spectrum. The slight mismatch between both curves, especially visible around the resonance wavelength, could be caused by surface and interface roughnesses that are not taken into account in our simple DBR modelling, as well as from spatial non-uniformity within the DBR layers. The good agreement between numerical and experimental results demonstrates the good quality of the SiC-based DBRs and the accuracy of the optical constants deduced by spectroscopic ellipsometry. The large values of reflectivity and stopband obtained with only 1.5 pairs are due to the high RI contrast (0.65 at 470 nm) between SiC and III-N layers, much larger than what is achievable between the III-N compounds. Consequently, the use of single-crystal SiC layers enables to fabricate DBRs on Si with a significant lower number of pairs and a larger stopband than with III-N based DBRs. Furthermore, no complex stress management is required for the monolithic growth of SiC-based DBR on Si resulting also to an even thinner structure, and thus a faster and cheaper growth. As uniformity is an important parameter in device manufacturing, reflectance mapping over the entire wafer was also measured and analyzed for the DBR B pictured in Fig. 5(a). Mappings of R_*max*_ and of λ(R_*max*_) are shown in Fig. 5(c,d), respectively. The average values are 52% and 430 nm for R_*max*_ and λ(R_*max*_), respectively, with standard deviations of ±3% and ±5 nm (i.e. ±1%) over the 100 mm-diameter wafer, respectively. Similar results were obtained between both DBR A and B. These values demonstrate a good uniformity of the DBR structures, with a deviation similar to what is obtained from the light emission of commercial GaN-based LEDs on Si^27^. It is also worth noting that thanks to their large stop-band, mismatch between the peak reflectance of a SiC-based DBRs and the LED light emission, often unavoidable with non-uniform growth, is less critical than with DBRs with much smaller stop-band as typically obtained with III-N DBRs.

### Electrical properties

**Figure 6:**
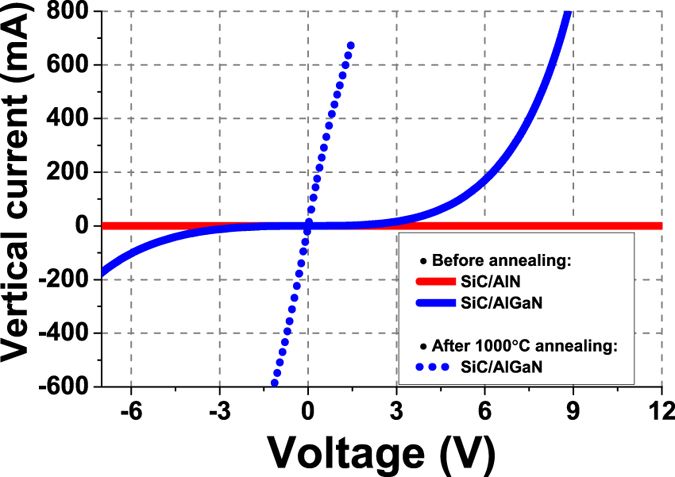
Room temperature vertical current versus applied voltage characteristics for the SiC/Al(Ga)N DBRs on Si, before (solid lines) and after annealing (dotted line) at 1000 °C under N_2_ atmosphere.

Vertical electrical conductivity of the SiC-based DBRs was also measured. Figure 6 shows the I-V characteristics of the DBRs before and after annealing. Before annealing, no significant current flow was measured from the DBR A for voltages up to 15 V, confirming that the AlN layer is an efficient current blocking layer which prevents vertical current injection despite the high electrical conductivity of the SiC layers. On the other hand, a high vertical current flow is detected for the DBR B, but with a strong rectifying behaviour due to the high energy barrier at the Ni/SiC interface (Schottky contact). This rectifying behaviour was fully suppressed after a rapid annealing of the Ni contacts at 1000 °C under N_2_ atmosphere as shown by the dotted line in Fig. 6, leading to a vertical current density as high as 70 A/cm^2^ at an applied bias of just 1.5 V (resulting to a total series resistance of only ~2Ω) which is sufficient to drive commercial HB-LEDs. These electrical results clearly demonstrate the high electrical conductivity of the SiC/AlGaN DBR structure and to the best of our knowledge, it is not only the first demonstration of an electrically conductive DBR compatible with GaN optoelectronic devices, but also the first electrically conductive single-crystal DBR grown on Si. It is also worth noting that the growth of single-crystal SiC on nitride compound could also be used to develop SiC-based photonic devices on Si operating in the visible and IR spectra^2^,^3^.

In summary, a new vertically-conductive DBR made of single-crystal SiC and AlGaN doped thin-films was successfully fabricated on Si substrate. The crack-free SiC-based DBR has very high optical uniformity with variation of 3% or less over the 100 mm Si wafer and shows a large stopband of 100 nm (at 80% of maximum reflectance). Thanks to the high contrast of refractive index of 0.65 over the entire visible spectrum, a high average maximum reflectance of 54% is achieved with only 1.5 pair centered in the blue region of the visible spectrum. Furthemore, high vertical conductivity was demonstrated with a measured current density of 70 A/cm^2^ for an applied bias of just 1.5 V, without optical degradation compared to a non-conductive SiC/AlN DBR counterpart. Those results show that single-crystal doped SiC/AlGaN DBR with high RI contrast, high electrical conductivity and high expected thermal conductance is a promising photonic structure for the large-scale integration of SiC and GaN-based visible optoelectronic devices on Si wafer, as well as for the development of optical micro-cavity devices on Si substrate.

## Methods

### DBRs Fabrication

Both DBR structures were grown on 100 mm-diameter and 525 μm-thick Si(111) wafers. In order to allow vertical current injection and to minimize the electrical resistivity, n^+^-type (Sb) doped Si wafers with a low resistivity of 0.01 to 0.02 Ω.cm were chosen. Each structure was designed to provide a maximum reflectance at 470 nm, matching the blue emission of standard commercial GaN-LEDs. The SiC absentee layer is deposited first on the Si substrates using a hot-wall vertical low-pressure chemical vapor deposition (LPCVD) reactor as described in previous works^23^,^28^. The heteroepitaxy of the SiC follows a two-step approach used to accommodate the large lattice-mismatch between Si and SiC (20%). This 2 steps approach consists of the creation of a buffer layer by carbonization of the silicon surface, then followed by the single-crystal SiC growth. The buffer layer, also called “carbonization layer” is formed by reaction of the silicon surface with a carbon-base gas^1^. For this work, the single-crystal SiC layer is grown at a temperature of 1000 °C using an alternating supply epitaxy (ASE), meaning that Si and C atoms are supplied in alternative pulses. In a previous work, ASE was found to provide better thickness uniformity, lower density of defects and better morphology than the standard concurrent supply epitaxy (CSE) growth^23^. We emphasize that because of the temperature limitation imposed by the melting point of Si at 1420 °C, only the cubic crystalline structure of SiC, called cubic polytype (or 3C-SiC), can be grown on Si^1^. The λ/4-thick nitride layers are deposited on top of the absentee layer using a standard MOCVD tool. Undoped AlN was used for the DBR A, while a Si doped Al-rich AlGaN layer with an expected carrier concentration of 5.10^18^ cm^−3^ was used for the DBR B. To terminate, a top λ/4-thick SiC layer was deposited with the same LPCVD tool as previously, completing the 1.5-pairs DBRs. Thanks to the low lattice-mismatch between Al(Ga)N and SiC, only a direct ASE deposition was used for this final deposition. Test samples similar to the DBR structures but with non-optimized layer thicknesses were also fabricated for optical and physical characterizations.

### DBRs Characterisation

Variable angle spectroscopic ellipsometry (J.A. Woollam VUV-VASE Gen-II ellipsometer) was used to deduce the dispersion of the complex refractive index and the layer thicknesses of the materials constituting the DBR samples. Those ellipsometric measurements were performed for at least three angles of incidence and over a spectral window spreading from 300 nm to 800 nm. Data were acquired after each layer deposition and several test samples with different layer thicknesses were used. Using several thicknesses enabled to verify that the fitting model was accurate enough to describe the optical properties of each material. The data were then fitted using the Woollam proprietary software WVASE, enabling to deduce complex refractive index and thickness of each layer. Transmission electron microscopy (TEM) and x-ray diffraction (XRD) were used to analyse the microstructure of the DBR samples. TEM measurements were carried out on cross-section using a FEI Tecnai F30 system (operating at 300 kV), while XRD was performed with a PANalytical Empyrean x-ray diffractometer, with a high resolution four-crystal Ge(220) asymmetrical incident beam monochromator, using CuKα radiation, and a PIXcel^3D^ detector with a fixed anti-scatter slit. 2θ-ω scan XRD acquisitions were done from 25° to 96° with an increment of 0.01°/step and a 0.1 s duration per step. Chemical analysis of the layers was investigated by X-ray photoelectron spectroscopy (Kratos Ultra photoelectron spectrometer) using AlK_*a*_ radiation over a scanning area of 700 μm × 300 μm. The XPS energy scale was calibrated using the C-C line bond at 284.6 eV and depth profiling of the chemical composition was achieved on selected test structures by combining *in-situ* Ar sputtering (at 4 keV) and XPS analysis at regular etch steps. Atomic force microscopy (Park NX20) was used in taping mode to acquire the samples topography over areas of 10 μm × 10 μm in size. Wafer curvature profiles pre- and post-DBR depositions were measured with a Tencor thin-film stress measurement system (model FLX-2320). Reflectance spectra of the DBR structures were measured at normal incidence with a FilmTek^*TM*^ 1000 M reflectometer equipped with an automated XY stage for mapping characterisation. Top-bottom current-voltage (I-V) characteristics of the DBR structures were measured at room temperature with an Agilent B1505A power device analyser connected to a probe station. In order to perform the electrical measurements, 300 nm-thick Al and Ni thin-films were deposited by DC sputtering (Surrey NanoSystems SNS Gamma PVD) on the DBR structures, respectively on the bottom side (Si surface) and top side (SiC surface). A shadow mask was used during the deposition of the Al contacts on Si to form cm-large contacts, while the deposited Ni film was patterned by photolithography followed by wet etching to form 1 mm^2^ square-shape contacts, equivalent in size to real commercial LEDs. To reduce the contact resistance with SiC, the Ni contacts were also annealed under N_2_ atmosphere at 1000 °C for 60 s using a rapid thermal processor tool (AG Associates Heatpulse 610).

## Additional Information

**How to cite this article**: Massoubre, D. *et al.* Vertically Conductive Single-Crystal SiC-Based Bragg Reflector Grown on Si Wafer. *Sci. Rep.* **5**, 17026; doi: 10.1038/srep17026 (2015).
